# Simultaneous integrated boost of intensity‐modulated radiation therapy to Stage II‐III non‐small cell lung cancer with metastatic lymph nodes

**DOI:** 10.1002/cam4.3446

**Published:** 2020-09-09

**Authors:** Qing‐Song Li, Na Liang, Wei‐Wei Ouyang, Sheng‐Fa Su, Zhu Ma, Yi‐Chao Geng, Wen‐Gang Yang, Yin‐Xiang Hu, Hui‐Qin Li, Bing Lu

**Affiliations:** ^1^ Department of Thoracic Oncology Affiliated Hospital of Guizhou Medical University Guiyang China; ^2^ Guizhou Cancer Hospital Guiyang China

**Keywords:** metastatic lymph nodes, nonsmall cell lung cancer, radiotherapy, SIB‐IMRT, stage II‐III

## Abstract

Local tumor failure remains a major problem after radiation‐based nonsurgical treatment for unresectable locally advanced nonsmall cell lung cancer (NSCLC）and inoperable stage II NSCLC. The aim of this study was to evaluate the feasibility of simultaneous integrated boost of intensity‐modulated radiation therapy (SIB‐IMRT) to stage II‐III NSCLC with metastatic lymph nodes (ChiCTR 2000029304). Patients were diagnosed by pathology or PET‐CT. PTV was divided into two parts as follows, the PTV of primary tumor (PTVp) and the PTV of metastatic lymph nodes (PTVn). The radiotherapy doses were simultaneously prescripted 78 Gy (BED = 101.48 Gy) for PTVp and 60‐65 Gy (BED = 73.6‐81.25 Gy) for PTVn, 26f/5.2 weeks. Response was scored according to WHO criteria. Radiotherapy toxicity was scored according to RTOG criteria. Hematology and gastrointestinal toxicity were scored according to CTCAE1.0 criteria. A total of 20 patients were enrolled. Seventeen patients were diagnosed by pathology and three patients were diagnosed by PET‐CT. All patients were treated with SIB‐IMRT. The objective response rate (ORR) was 90%, with CR 25%, PR 65%, NC 10%, and PD 0%. Although radiotherapy toxicity was common, there were no grade ≥3, with radiation pneumonitis (10 cases), esophagitis (17 cases), and dermatitis (12 cases). The local control rates at 1, 3, and 5 years were 85%, 75%, and 70%, respectively. The overall survival（OS）and local progression‐free survival (LPFS) rates at 1, 3, and 5 years were 90%, 42.6%, and 35.5% and 84.4%, 35.5%, and 28.4%, respectively. SIB‐IMRT can significantly improve ORR and survival for stage II‐III NSCLC with metastatic lymph nodes, with high safety, and satisfactory efficacy. However, due to the limitation of small sample, these findings are needed to confirm by future trials with a larger sample size.

## Graphical abstract

The different radiation doses were simultaneously prescripted 78 Gy (PTVp）and 60‐65 Gy (PTVn). SIB‐IMRT can significantly improve ORR and survival for stage II‐III NSCLC with metastatic lymph nodes, with high safety and satisfactory efficacy.

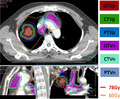

## 1 INTRODUCTION

Concurrent thoracic chemoradiotherapy (CCRT) is the standard of care in patients with good performance status and inoperable stage II‐III nonsmall cell lung cancer (NSCLC) who have metastatic lymph nodes, especially for locally advanced NSCLC.^1^ The radiation dose is one of the controversial focuses. At present, the uniform dose of radiation therapy was 60 Gy, but treatment outcomes remain poor. RTOG 0617 demonstrated a detrimental effect of dose escalation with worse overall survival. But there were several limitations to this study. Radiotherapy techniques included 3DCRT (53%) and IMRT (47%).^2^ There were no 4D simulation and adaptive techniques. Radiotherapy planning was not strictly observed dose‐volume guidelines for the heart.^2^ Radiotherapy techniques and heart dose were associated with overall survival.^3^, ^4^, ^5^ Radiotherapy dose (74 Gy, BED = 84 Gy） failed to achieve a radical dose (BED ≥ 100 Gy^6^） and may also bring more toxicity. The poorer results with 74 Gy were probably caused by a combination of these factors. However, it is not denied the relationship between radiotherapy dose and biological effect. RTOG trials led to the conclusion that local tumor control was significantly correlated with improving survival.^7^ So 60 Gy may be not a reasonable dose^2^, ^8^, ^9^ and further study of radiation high dose is necessary. In this study, it is conceived that using the biological effective dose^10^ (BED) ≥ 100 Gy of large fractionated radiotherapy for stage Ⅰ‐Ⅱ NSCLC can obtain more than 90% local control rate^6^ and recurrence rate of mediastinal metastatic lymph nodes for locally advanced NSCLC with concurrent chemoradiotherapy (median dose 60 Gy/30f) was only 20%.^11^ Different doses of radiotherapy were simultaneously prescribed for primary tumor and metastatic lymph nodes through SIB‐IMRT. The aim of this study was to evaluate the safety and efficacy of SIB‐IMRT for stage II‐III NSCLC with metastatic lymph nodes.

## 2 MATERIALS AND METHODS

### 2.1 Patients, study design, and treatment

Inclusion criteria were as follows: (a) histologically or cytologically or PET‐CT confirmed NSCLC; (b) newly diagnosed stage II‐III disease (staged according to the 2009 system of the American Joint Committee on Cancer); (c) KPS ≥ 70; (d) no contraindications to radiation therapy or chemotherapy; (e) normal range of blood routine and biochemical testing(f) good compliance for treatment and follow‐up.

### 2.2 Pretreatment evaluations

All patients underwent fiberoptic bronchoscopy and contrast‐enhanced computed tomography (CT) of the chest to evaluate the extent of the primary tumor and regional lymph node status. All patients also underwent bone scintigraphy, contrast‐enhanced CT of the abdominal region, and magnetic resonance imaging (MRI) of the brain to detect distant metastases. Positive findings on positron emission tomography (PET)/CT or bone scintigraphy required other additional radiologic confirmation (eg, MRI or CT of bone). Pretreatment evaluations were to be completed within 2 weeks before treatment was begun.

### 2.3 Thoracic radiotherapy

Intensity modulated radiation therapy (IMRT) were created using the Pinnacle (ADAC Laboratories, Milpitas, CA) treatment planning system. the patient was positioned in the supine position with thermoplastic film fixation and 5‐mm‐thickness enhanced computer tomography (CT）. IGRT (CBCT) was used in all patients. Considering the possibility of tumor shrinkage, CT was performed again when the radiation dose reached 36‐39 Gy/12‐13 f. CT imagings were fused to determine whether RT planning should be adjusted.

The target volume of primary tumor and metastatic lymph nodes were delineated, respectively. The gross tumor volume of primary tumor (GTVp) included the thoracic primary tumor in lung windows and was outlined on the treatment planning CT scan, the clinical target volume of primary tumor (CTVp) was defined as the GTVp plus a 0.6‐cm margin with anatomical correction, the planning target volume of primary tumor (PTVp) was defined as the CTVp plus another margin of 0.5 to 1.0 cm. The gross tumor volume of metastatic lymph (GTVn) included any enlarged (>1 cm on short axis) metastatic lymph nodes and was outlined on the treatment planning CT scan, the clinical target volume of metastatic lymph (CTVn) was modified by the GTVn and expanded outward by 0.6 cm combined with anatomical correction, the planning target volume of metastatic lymph (PTVn) was defined as the CTVn plus another margin of 0.5 cm.

**FIGURE 1:**
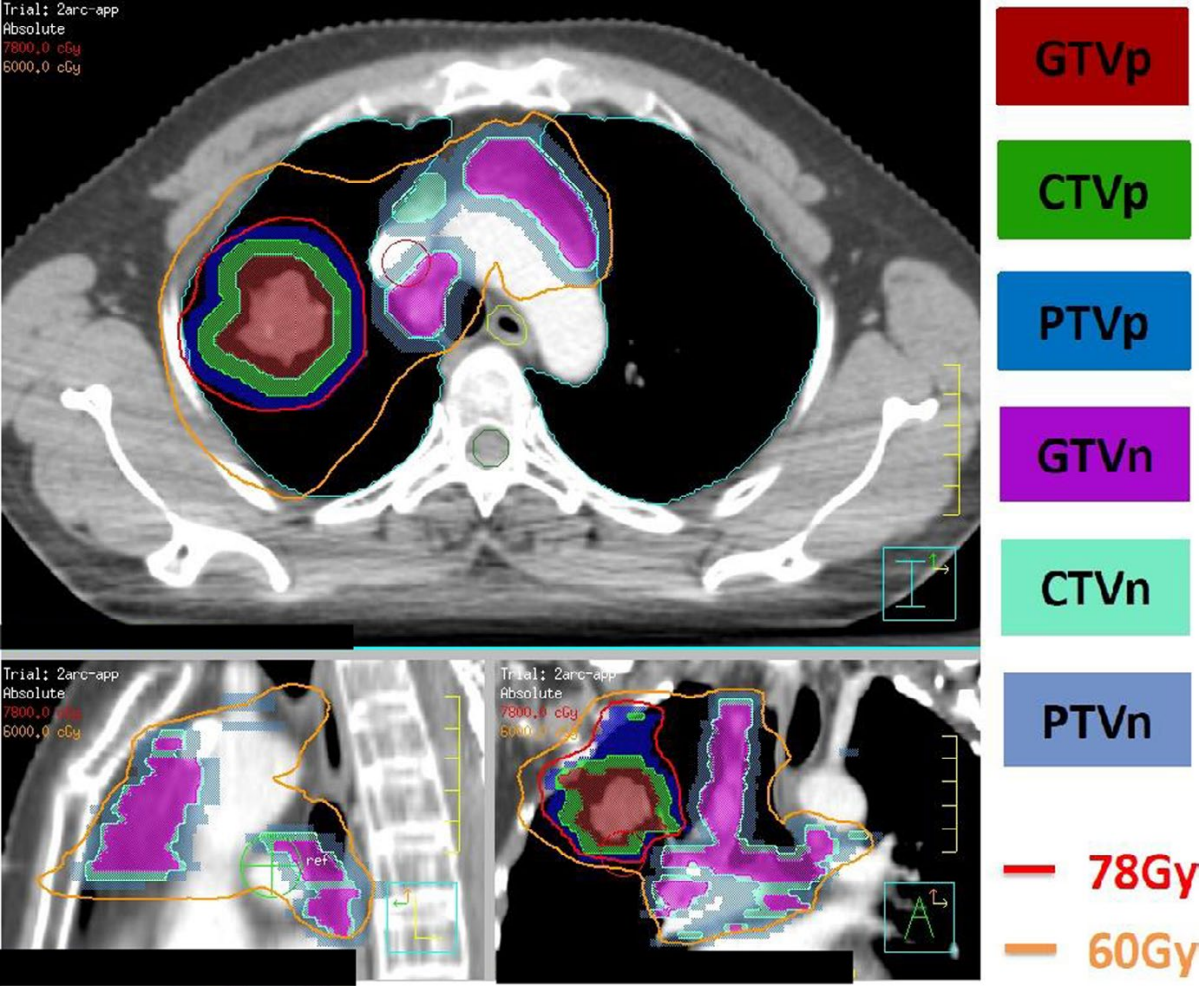
The radiotherapy doses were simultaneously prescripted for PTVp and PTVn

**FIGURE 2:**
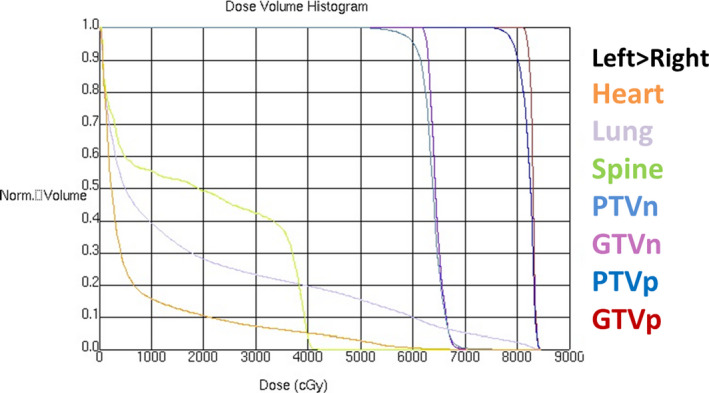
Dose volume histogram

Different doses of radiotherapy were simultaneously prescribed for PTVp and PTVn through SIB‐IMRT. The radiotherapy doses were 78 Gy (BED = 101.48 Gy) for PTVp and 60‐65 Gy (BED = 73.6‐81.25 Gy) for PTVn, 26f/5.2 weeks, respectively. The radiotherapy plan was evaluated as 95% of the prescription dose line including 95% of PTV. The percentage of total lung volume receiving ≥20 Gy （V20）, whole lung dose (MLD), and mean heart dose (MHD) was to be kept at ≤32%, ≤20 Gy and ≤30 Gy respectively (Figures 1, 2).

#### 2.3.1 Chemotherapy

Platinum‐based doublet chemotherapy (cisplatin in combination with docetaxel, paclitaxel, or pemetrexed) or single drug was given every 21 to 28 days. The chemotherapy regimens of all patients were as follows: one case of docetaxel, one case of pemetrexed, one case of cetuximab+docetaxel, one case of pemetrexed+cisplatin, one case of paclitaxel liposome+carboplatin, one case of pemetrexed+cisplatin, one case of paclitaxel liposome+carboplatin, nine cases of docetaxel+cisplatin (two cases of docetaxel+cisplatin+Endostar）. Chemotherapy cycles were from 2 to 4. Four patients refused chemotherapy.

### 2.4 Evaluation of treatment‐related toxicity and response

Response was scored according to WHO criteria as follows: complete response (CR), partial response (PR), and no change (NC), progressive response (PD). Radiotherapy toxicity was scored according to RTOG criteria. Hematology and gastrointestinal toxicity were scored according to CTCAE1.0 criteria.

### 2.5 Follow‐up evaluations and Statistical analyses

At 1 month after completion of treatment, patients underwent CT scanning of the chest and abdominal region and MRI of the head to assess tumor response. These tests were then repeated every 3 months for 2 years and every 6 months thereafter. Primary endpoints were overall survival (OS), local progression‐free survival (LPFS) and acute toxicity. Kaplan‐Meier analyses were used for statistical analysis.

## 3 RESULTS

### 3.1 Patient characteristics

From December 2009 to March 2019, 20 patients were enrolled in this study. There were 3 patients with stage Ⅱ A, 1 patient with stage Ⅱ B, 12 patients with stage Ⅲ A, and 4 patients with stage Ⅲ B. The patients with stage II refused operation because of old‐aged patients (three cases) and pulmonary dysfunction (one case). There were 17 males and 3 females. Median age of patients was 75 years (range, 44‐82 years). Fifty percent of patients (10/20) were older than 70 years. Age of 8 patients was ≥77 years (range, 77‐82 years). There were 4 adenocarcinomas, 10 squamous cell carcinomas, 3 nonsmall cell lung carcinoma, and 3 clinically diagnosed lung cancers. Median long axis of primary tumor was 4.55 cm (range, 1.2 −11 cm). Median number of metastatic lymph nodes was 1 (range, 1 −5). Median volume of GTVp was 69.99 cm^3^ (range, 8.74‐530.07 cm^3^). Median volume of PTVp was 189.22 cm^3^ (range, 83.85‐886.71 cm^3^). Median volume of GTVn was 35.96 cm^3^ (range, 4.37 −139.05 cm^3^). Median volume of PTVn was 148.37 cm^3^ (range, 35.79‐405.68 cm^3^). The number of patients with V 20 ≤ 20%, 21%‐25%, 25%‐30%, and >30% were 4, 7, 7, and 2 (range, 12.1%‐32%, median 24.6%), respectively. The number of with MLD ≤ 20 Gy and >20 Gy were 17 and 3 (range 8.57‐21.23 Gy, median MLD 16.67 Gy), respectively. The number of patients mean heart dose (MHD) ≤26 Gy and >26 Gy were 16 and 4 (range 1.83‐32.41 Gy, median 7.65 Gy), respectively (Table 1）.

**TABLE 1:** Clinical characteristics, treatment data and survival of 20 patients of stage II‐III NSCLC

No	Sex	A	B	C	D	stage	metastatic lymph node		GTV_P_ (cm^3^)	PTV_P_ (cm^3^)	GTV_N_ (cm^3^)	PTV_N_ (cm^3^)	V20 (%)	MLD (Gy)	MHD (Gy)	PD	Death	Cause of death	OS (month）	LPFS (month）
							Drainage area	Number												
1	Male	+	−	−	−	T2aN2M0	4R + 10R	2	79.13	290.08	46.93	169.93	24.9	18.32	11.91	L + M	Yes	L + M	64	62
2	Male	+	−	−	−	T3N2M0	4L + 7	2	465.91	886.71	37.28	137.57	31.3	20.43	32.41	L	Yes	L + Hemoptysis	23	23
3	Male	−	−	−	−	T1aN2M0	5 + 7	5	94.85	360.35	72.14	281.72	24.3	13.73	6.3	No	Yes	Pulmonary infection	12	12
4	Male	+	−	−	−	T2aN1M0	10L	1	26.13	123.83	61.65	239.53	32	21.19	30.86	No	No		82	82
5	Male	−	−	+	+	T2bN1M0	10R	1	49.2	163.01	16.03	63.07	19.9	14.96	2.35	No	Yes	myocardial infarction	43	43
6	Male	−	+	+	−	T3N1M0	10L	1	93.83	234.75	71.32	201.32	25.5	17.92	22.79	L	Yes	Sudden cardiac death	14	12
7	Male	−	−	+	−	T2aN1M0	10L	1	27.53	83.85	22.37	84.38	16.1	10.96	9.04	No	Yes	Sudden cardiac death	24	24
8	Female	−	+	−	−	T3N2M0	4R	1	398.47	711.64	32.44	123.93	26.5	16.96	19.39	M	Yes	Pulmonary infection	18	9
9	Male	+	−	−	−	T3N2M0	4R	1	76.35	204.29	62.72	159.16	23.9	21.23	7.58	No	No		48	48
10	Male	+	−	−	−	T3N2M0	2R	1	70.11	211.57	10.1	72.11	22.5	14.32	4.1	No	No		43	43
11	Male	+	−	−	−	T3N1M0	10L（ fusion）	Fusion	530.07	868.64	107.51	261.05	22.8	17.7	26.92	No	Yes	Respiratory failure	15	15
12	Male	+	+	−	−	T3N3M0	4L	1	69.87	174.15	21.56	74.76	27.3	16.49	7.7	No	No	Pulmonary infection	16	16
13	Male	−	−	−	−	T2N2M0	4R	1	26.22	111.72	4.37	44.54	12.11	8.57	6.84	No	No		24	22
14	Female	−	+	−	−	TN1N1M0	10L	1	8.74	84.70	58.20	227.79	28.00	14.17	30.9	L + M	No		66	30
15	Male	−	+	−	−	T3N2M0	4R	2	46.28	133.98	7.68	35.79	17.9	18.32	1.83	No	Yes	Pulmonary infection	32	32
16	Male	−	−	−	−	T4N3M0	4R/L/7/10R	5	231.13	475.15	34.01	188.11	25.28	16.96	8.48	No	No		16	16
17	Female	−	+	−	+	T2N3M0	4R/L/5/6	6	52.12	96.08	12.15	76.36	20.49	12.78	1.98	No	No		14	14
18	Male	+	−	−	−	T4N2M0	6/7	5	261.14	472.17	34.63	132.15	21.92	13.82	4.74	L	Yes	L	15	13
19	Male	−	−	−	−	T3N2M0	2R/3A/4R/5/10R	5	35.24	118.53	80.61	245.22	27.43	16.85	5.94	No	No		13	8
20	Male	−	−	−	−	T2N2M0	2R/3A/4R(fusion), 7	3	23.9	87.08	139.05	405.68	25.80	15.34	7.60	No	Yes	Pulmonary infection	6	6

Note

A: chronic obstructive pulmonary disease, B: hypertension, C: coronary heart disease, D: diabetes mellitus, L, local recurrence; M, distant metastasis.

### 3.2 Response

The CR, PR, and SD rate was 25% (5 cases）, 65% (13 cases）, and 10% (2 cases）, respectively. The CR, PR, and SD rate of primary tumor was 30% (6 cases）, 55% (11 cases）, 15% (3 cases）, respectively. The CR, PR, and SD rate of metastatic lymph nodes was 25% (5 cases）, 60% (12 cases）, 15% (3 cases）. The clinical benefit rate was 100%.

### 3.3 Progress, cause of death, and survival

**FIGURE 3:**
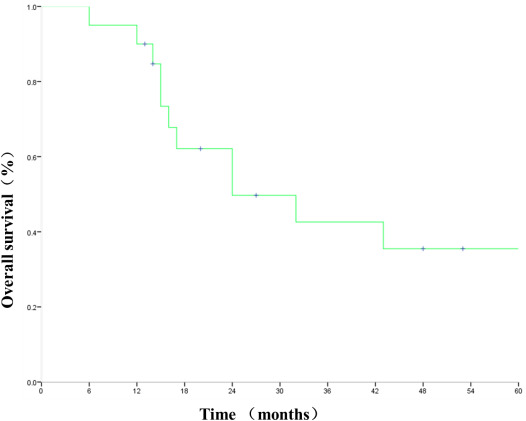
Overall survival for all patients

**FIGURE 4:**
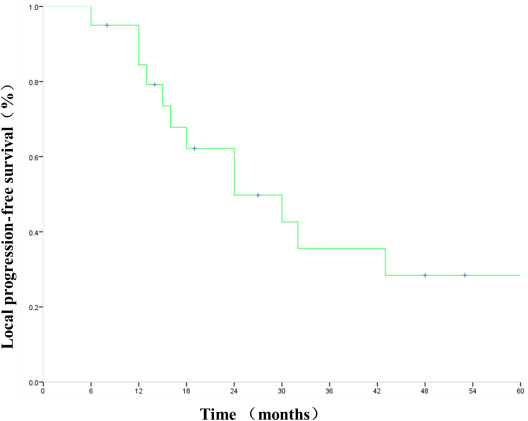
Local progression‐free survival for all patients

During follow‐up, there were local recurrence (primary tumor failure in two patients, regional lymph node failure in three patients) and/or metastasis progressed in seven patients (two patients with local recurrence and bone metastasis, one patient with local recurrence and pulmonary metastasis, one patient with local recurrence, one patient with local recurrence and hemoptysis, one patient with alone lung metastasis, one patient with malignant pleural effusion). After disease progress, none of the patients accepted PD‐1 testing and three patients accepted EGFR/ALK/ROS testing. One patient was treated with gefitinib because of the positive result. Two patients were treated with second‐line chemotherapy. The rest patients refused follow‐up treatment. By the last follow‐up, 12 patients died. The cause of death in nine patients was not related to tumor (three died of cardiogenic death, five died of pulmonary infection, and one died of respiratory failure). The cause of death in three patients was related to tumor (one died of local recurrence with metastasis, one died of local recurrent hemoptysis, and one died of local recurrence）. The local control rates at 1, 3, and 5 years were 85%, 75%, and 70%, respectively. The OS and LPFS rates at 1, 3, and 5 years were 90%, 42.6%, and 35.5% and 84.4%, 35.5%, 2 and 8.4%, respectively (Figures 3, 4). The mean survival time (MST) was 24 months.

### 3.4 Toxicity

No patient experienced grade 3+ acute radiotherapy toxicity (Table 2）. Grade Ⅰ radiation pneumonitis was observed in nine patients and grade Ⅱ in one patient. Grade Ⅰ radiation dermatitis was observed in nine patients and grade Ⅱ in three patients. Grade Ⅰ radiation esophagitis was observed in seven patients and grade Ⅱ in 10 patients. Radiation pulmonary fibrosis was observed in one patients, which occurred 3 months after radiotherapy (the serial number 12, lung V20 was 27%, MLD was 16.49 Gy). Grade Ⅰ, Ⅱ, Ⅲ, and Ⅳ of gastrointestinal and hematological toxicity were 6, 8, 1, and 0 and 1, 6, 5, and 4 cases, respectively, all of which occurred in concurrent chemoradiotherapy.

**TABLE 2:** Radiotherapy toxicity of 20 patients of stage II‐III NSCLC

	Grade	Radiation pneumonitis	Radiation dermatitis	Radiation esophagitis	Radiomyelitis	Heart injury
All patients		10	12	17	0	0
	1	9	9	7		
	2	1	3	10		
Radiotherapy alone		2	1	4		
	1	2	1	4		
	2	0	0	0		
Chemoradiotherapy		8	11	13		
	1	7	8	3		
	2	1	3	10		

## 4 DISCUSSION

At present, the radiotherapy dose ≥ 60 Gy/30 f is needed for concurrent chemoradiotherapy to locally advanced NSCLC,^12^ but the local control rates at 1, 3, and 5 years were about 70%, 50%, and 40%,^13^ and the OS rate at 5 years was about 15%.^12^, ^14^ The local recurrence rates of stage Ⅱ‐Ⅲ NSCLC with concurrent chemoradiotherapy at 1, 2, 3, and 5 years were 23%,^15^ 30.8% −37.1%,^4^ 28.1%, and 28.9.^14^ Uncontrolled local tumors may be a potential source of distant metastasis.^16^ Although the RTOG0617 study showed that 60 Gy may be more reasonable than other radiotherapy dose,^2^ further analysis of the study showed that the result was affected by many other factors.^4^ There is still debate about that increasing the dose of radiotherapy can reduce recurrence and prolong survival. A phase II clinical study by KongFM et al showed that the local control rate was 82% at 2 years and 30% at 5 years because of the high radiotherapy dose (86 Gy/30 day) with adaptive radiotherapy guided by PET/CT.^8^ Many studies still supported the idea that high dose of radiotherapy was necessary.^17^, ^18^, ^19^, ^20^ After the report of RTOG0617 study in 2012, Machtay et al conducted a meta‐analysis, which included seven prospective randomized concurrent chemoradiotherapy studies about locally advanced NSCLC. The study^21^ showed that BED ≥74.67 Gy (conventional fractionated dose 62‐64 Gy) was more beneficial to improve the local control and survival, increasing the time‐adjusted BED (tBED) of 1 Gy can increase the local control by 3%, and the BED of 1 Gy can increase OS by 4%. After conventional fractionated radiotherapy, dose escalation for local tumor may improve local control and survival.^22^

Through changing the fractionated radiotherapy dose of 2.5‐6 Gy with concurrent chemoradiotherapy for locally advanced NSCLC, the CR, PR, 1, 2, 3 years OS rates, and MST were 26.5%, 42.9%, 63.3%, 40.8%, 20.4%, and 22 months, respectively.^23^ A fraction dose of 3 Gy and total dose of 65‐68 Gy were given after initially 50 Gy/20 fractions, the 3‐years OS and progression‐free survival (PFS) rates were 32.1% and 29.8%, respectively, and the 1‐, 2‐, and 3‐years LRPFS rates were 69.6%, 60.9%, and 60.9%, respectively.^24^ Based on the characteristics of IMRT, the dose of the PTV was kept at 60 Gy and the dose of GTV was 72‐78 Gy synchronously, the result showed that MST was 25.3 months.^13^ 63‐103 Gy was the recommended radiotherapy dose to improve the local control rate.^25^ The commonness of these studies was that the primary tumor and drainage lymph nodes were defined as the same target. The ways to increase dose of physical or BED were as follows: dose escalation after conventional fractionated radiotherapy, different doses were simultaneously prescribed to GTV and PTV. The aim was to improve the tumor local control and survival. The way of increasing the dose of radiotherapy in our study was different from the above studies. The primary tumor and drainage lymph nodes were defined as different targets (GTVp and GTVn). Different doses of radiotherapy were simultaneously prescribed for primary tumor and metastatic lymph nodes through SIB‐IMRT. One principle is that the BED ≥ 100 Gy for stage I NSCLC can obtain a local control rate of >90%,^26^ significantly reduce the local recurrence rate and improve the survival rate at 3 years.^27^ Another principle is that the long‐term local control rate is more than 50% through conventional fractionated radiotherapy dose (60‐66 Gy) for mediastinal metastatic lymph nodes.^28^ Through SIB‐IMRT, the radiotherapy doses were simultaneously prescripted 78 Gy (BED = 101.48 Gy) for primary tumor and 60‐65 Gy/26 f (BED = 73.6‐81.25 Gy) for metastatic lymph nodes.The result showed that the ORR was 90% (18/20), and the SD was only observed in two patients (primary tumor reduced by 14%, 30%, metastatic lymph node reduced by 44%, 32%). The objective response rate (ORR）of primary tumor and metastatic lymph node was both 85% and there was no PD. The local control rates at 1, 3, and 5 years were 85%, 75%, and 70%, respectively. Only four patients were diagnosed as local recurrence confirmed by imaging and/or pathology and three of them died from the following causes: myelosuppression grade IV, sudden cardiac death, hemoptysis. The result showed that the ORR was kept at 90% by SIB‐IMRT, which was superior to the result of chemotherapy and conventional fractionated radiotherapy for local advanced NSCLC.^29^ It may be due to the increase of ORR, the proportion of tumor shrinkage and regression increased, which was beneficial to reduce the recurrence and mortality rate and positively correlated with the prolongation of survival rate.^8^ The OS and LPFS rates at 1, 3, and 5 years were 90%, 42.6%, and 35.5% and 84.4%, 35.5%, and 28.4%, respectively. The MST was 24 months.

The injury control index used in the evaluation of radiotherapy plan was based on the standard of conventional fractionated radiotherapy of locally advanced NSCLC. Acute radiation pneumonitis, radiation esophagitis, and radiation dermatitis were all grade 1‐2. Further analysis showed that grade 2 toxicity mainly occurred in concurrent chemoradiotherapy, which increased acute radiotoxicity.^14^ Compared with the conventional radiotherapy, there was no increase in radiotoxicity despite the increase in fractionated dose (3 Gy/f) and BED.^30^ The main systemic toxicity was gastrointestinal toxicity (grade 1 in six patients, grade 2 in eight patients, and grade 3 in one patient) and hematological toxicity (grade 2 in six patients, grade 3 in five patients, and grade 4 in four patients). All of them occurred in concurrent chemoradiotherapy. Radiotherapy alone had no obvious systemic toxicity. It showed that systemic toxicity was mainly related to cytotoxic drugs.

A total of 12 patients died. Five patients died of pulmonary infection (death time: 16, 6, 12, 18, 32 months after treatment; age: 55, 71, 78, 79, 85 years; V20: 26. 5%, 25.8%, 24.3%, 27.3%, 17.9%; RP grade:2, 0, 0, 2, 0; MLD < 20 Gy). Radiation pneumonitis (RP) takes place usually within 1‐6 months（mostly with 3 months）after completion of radiation therapy.^31^, ^32^, ^33^, ^34^, ^35^, ^36^, ^37^ MLD, V20, and V30 have been identified as the dosimetric factors most associated with RP.^38^, ^39^, ^40^, ^41^, ^42^, ^43^ The 2 years incidence of grade ≥2 RP was 7% with a V20 of 22% to 31% (*P* = .0013).^38^ When MLD was <20 Gy, the incidence of RP was 8%.^38^ From time factor, dosimetric factors, and pulmonary infection (diagnosed by CT Imaging and related inspection result), pulmonary infection was not associated with RT and did not overlay RP. According to Chest‐CT, pulmonary infection did not overlay primary tumor. Three patients died of cardiogenic diseases (age 79, 78, 80 years； accompanied by hypertension and diabetes, coronary heart disease with coronary stent implantation, paroxysmal atrial fibrillation; MHD: 2.35 Gy, 22.79 Gy, 9.04 Gy). Four patients died of local recurrence, local recurrence along with metastasis, local recurrence with massive hemoptysis and respiratory failure, respectively.

SIB‐IMRT has high safety and satisfactory efficacy for stage II‐III NSCLC with metastatic lymph nodes. The duration of radiotherapy was reduced by at least a week. We acknowledge several limitations to the current study. This was a small sample study (20 patients). PET‐CT was not routinely performed for patients, which reduced the accuracy of N status. 4D simulation and adaptive radiotherapy technology was not routinely performed for patients. Considering limitations to the current study, these results must be confirmed by future trials with a larger sample size, which used modern imaging and radiotherapy techniques.

## 5 CONCLUSIONS

SIB‐IMRT can significantly improve ORR and survival for stage II‐III NSCLC with metastatic lymph nodes. This treatment approach has high safety and satisfactory efficacy. However, due to the limitation of small sample, these findings are needed to confirm by future trials with a larger sample size.

## CONFLICT OF INTEREST

All authors have read and approved the final manuscript. None of the authors have any financial disclosures or conflict of interest to declare.

## AUTHORS' CONTRIBUTIONS

BL designed the study. QSL, NL, WWO, SFS, ZM, YCG, WGY, YXH, HQL collected the data. BL and QSL undertook the data analysis and interpretation, and wrote the report. BL and QSL carried out the statistical analysis. All authors read and approved the final manuscript.

## ETHICAL APPROVAL

This study was reviewed by the ethical review boards in China (Ethics Committee of Guizhou Cancer Hospital, GuiYang, China). Informed consent for treatment was obtained from patients.

## CONSENT FOR PUBLICATION

Its publication has been approved by all coauthors. I would like to declare on behalf of my coauthors that the work described was original report that has not been published previously, and not under consideration for publication elsewhere.

## Data Availability

http://www.chictr.org.cn/edit.aspx?pid=23437&htm=4.
